# Early Identification of Residual Tumors following Microwave Ablation Using Contrast-Enhanced Ultrasonography in a Rabbit VX2 Liver Cancer Model

**DOI:** 10.1155/2020/2462058

**Published:** 2020-09-26

**Authors:** Huiming Yi, Baohuan Cai, Xi Ai, Kaiyan Li, Pengfei Song, Wei Zhang

**Affiliations:** ^1^Department of Medical Ultrasound, Tongji Hospital, Tongji Medical College, Huazhong University of Science and Technology, Wuhan City, Hubei Province, China; ^2^Department of Electrical and Computer Engineering, University of Illinois at Urbana-Champaign, Urbana, Illinois, USA; ^3^Beckman Institute for Advanced Science and Technology, University of Illinois at Urbana-Champaign, Urbana, Illinois, USA; ^4^Department of Pediatrics, Tongji Hospital, Tongji Medical College, Huazhong University of Science and Technology, Wuhan City, Hubei Province, China; ^5^Department of Hepatic Surgery, Tongji Hospital, Tongji Medical College, Huazhong University of Science and Technology, Wuhan City, Hubei Province, China

## Abstract

**Objective:**

It is difficult to evaluate the ablation effect immediately after thermal ablation of liver cancer by clinical imaging methods, due to the immediate formation of an annular inflammatory reaction band (IRB). This study is aimed at exploring the early identification indicators of the IRB and residual tumor postmicrowave ablation (MVA) using contrast-enhanced ultrasonography (CEUS).

**Methods:**

MVA was used to inactivate part of the tumor nodules in rabbit VX2 liver cancer models, leading to the coexistence of the IRB with residual tumors. Quantitative analysis of the perfusion parameters of the tumor and ablation zone was performed using CEUS, followed by liver biopsy and VEGFR-2 immunohistochemical staining.

**Results:**

All rabbits successfully tolerated VX2 tumor inoculation and MVA operation. No statistically significant difference existed between the IRB vs. residual tumors, the IRB vs. junctional areas, and residual tumors postablation vs. VX2 tumors before ablation in regional blood volume, blood velocity, and blood flow estimated by parameters *A*, *k*, and *A*^∗^*k* of CEUS quantitative analysis. There was a statistically significant difference between the IRB and normal liver parenchyma in regional blood velocity and blood flow (*p* = 0.005 and *p* = 0.023, respectively). Normal liver parenchyma showed nonspecific VEGFR-2 staining, while VX2 tumor before ablation and residual tumor after ablation both showed positive VEGFR-2 staining; the necrosis zone showed negative staining by VEGFR-2 immunohistochemical staining.

**Conclusion:**

MVA had no significant effect on the residual tumor hemodynamics. The blood flow in the IRB increased significantly as compared to normal liver parenchyma, resembling tumor hemodynamic patterns. CEUS can detect residual tumors immediately postablation only when they protrude from the annular-shaped IRB. In addition, VEGFR-2 targeted CEUS may have a great potential for detecting residual tumor after thermal ablation of hepatocellular carcinoma.

## 1. Introduction

Hepatocellular carcinoma (HCC) is one of the most commonly diagnosed malignant tumors in the world, with a globally increasing incidence rate [1]. With the total treatment effect, the thermal ablation of HCC is becoming more and more extensively used in the clinic [2]. The local thermal ablation can achieve complete inactivation of HCC nodules with a diameter of less than 3 cm. For single HCC nodules with a diameter of less than 2 cm, local thermal ablation may be the preferred treatment, and for single HCC nodules with a diameter of 2-3 cm, local thermal ablation is comparable to surgical resection [3, 4]. For patients with HCC who cannot be surgically resected, thermal ablation therapy could also be used for the local control of the tumor and preparing for transplantation [5]. Microwave ablation (MVA), as an important technique of tumor thermal ablation, is widely used in clinics [6].

Although the curative effect of MVA for localized HCC has been demonstrated, due to the heterogeneous spatial distribution, the irregular shape of the tumor, the local heating effect caused by blood diffusion, and operator dependence, tumor tissue may still remain activated in the ablation area, leading to tumor recurrence in a short term [7]. Therefore, it is very important to detect residual tumors early and provide supplemental treatment after ablation. However, based on the current clinical practice, postablation evaluation is typically conducted by contrast-enhanced CT or MRI 1 month after ablation [8]. None of the existing imaging methods can accurately evaluate the ablation effect immediately after ablation, due to the immediate formation of an annular inflammatory reaction band (IRB) around the lesion that lasts 2-6 months after MVA [9]. The annular-shaped IRB around the ablation zone is significantly enhanced during the arterial period, which resembles the residual tumors in the ablation area when evaluated by contrast-enhanced imaging techniques. Therefore, the conventional contrast-enhanced CT and MRI are limited in their abilities to early differentiate residual tumors from IRB postthermal ablation of HCC [10].

Contrast-enhanced ultrasonography (CEUS) can provide real-time, continuous dynamic imaging of a lesion from the beginning of wash-in to the end of wash-out, from which time-intensity curves and quantitative perfusion parameters can be derived [11]. CEUS is a potentially valuable technique in evaluating the efficacy of thermal ablation therapy, which compares favorably with CE-MDCT and MRI at the 1-month follow-up assessment [12, 13]. The abnormal nodular hypervascular region within the ablation zone would be identified as the residual viable tumor on CEUS; however, hyperemia IRB could bring substantial challenges to the identification of residual tumor immediately postablation [14].

In this study, a rabbit model of VX2 liver cancer was used as a preclinical model of HCC, and an MVA technique guided by ultrasound was used to ablate part of the tumor nodules in order to establish an animal model with the presence of the IRB and residual tumors coexisting, and the contrast-enhanced ultrasound technique was used to quantitatively analyze the patterns and perfusion parameters of the IRB and residual tumors in order to explore the early identification indicators of residual tumors.

## 2. Materials and Methods

### 2.1. Animals

This study was approved by the Animal Use and Care Committee of Tongji Hospital, Tongji Medical College, Huazhong University of Science and Technology (China). All New Zealand white rabbits, aged from two to three months and weighing from 2 to 3 kg, were obtained from the Experimental Animal Center of Tongji Hospital.

### 2.2. Rabbit VX2 Liver Tumor Model

The VX2 tumor fragments provided by the Experimental Animal Center of Tongji Hospital were implanted into the livers of 10 healthy New Zealand white rabbits. All rabbits were anesthetized via intramuscular injection of ketamine injection (40 mg/kg, intramuscular, Sigma-Aldrich, Inc., St. Louis, USA) and xylazine injection (5 mg/kg, intramuscular, Sigma-Aldrich, Inc., St. Louis, USA). The tumor inoculation process includes the following: take the subxiphoid area for the percutaneous puncture, penetrate a 16-gauge needle into the left lobe parenchyma of the rabbit liver guided by ultrasonography, then put a 1 mm^3^ VX2 tumor fragment into the needle flushed by 1 ml saline, and finally pull out the needle and gently compress the puncture area for 3 min after confirming the tumor fragment was flushed into the liver parenchyma. After 14 days of tumor inoculation, rabbit liver tumors with sizes of 1.71 ± 0.20 cm × 1.28 ± 0.29 cm were established according to our preliminary study [15].

### 2.3. Ultrasound-Guided MVA

**Figure 1:**
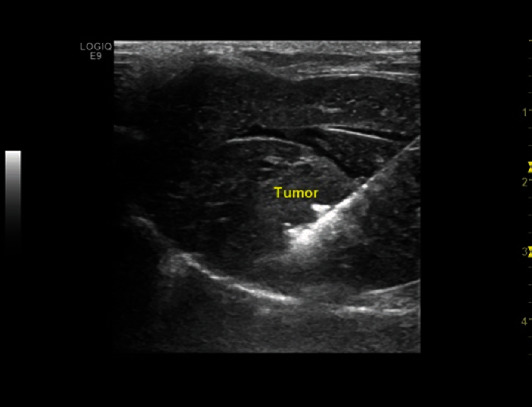
Microwave ablation of VX2 liver tumor in rabbits. A microwave ablation needle was inserted into the edge of the tumor nodule guided by ultrasound, ablating part of the tumor.

Microwave ablation was performed by a microwave unit (ECO 100AL1, Yigao Microwave System Inc., Nanjing, China) and a 16-G MVA needle with a 3 mm active tip length. In order to create a model of residual tumor following MVA, only the peripheral portion of the tumor and the surrounding normal tissue were ablated, leaving part of the tumor untreated (Figure 1). To carry out the project, the MVA needle was inserted into the edge of the tumor guided by ultrasound, and each tumor was treated for 3 min at 30 W. All the rabbits were anesthetized with an intramuscular injection of ketamine and xylazine mentioned above before microwave ablation.

### 2.4. Ultrasound Imaging Protocol

Each rabbit's lower abdomen skin was shaved to facilitate ultrasonic imaging. A GE LOGIQ E9 (GE Healthcare, Wauwatosa, WI, USA) equipped with a 7 MHz linear array transducer was used for CEUS and dynamic images recording. SonoVue (Bracco Imaging, Milan, Italy), a second-generation contrast agent composed of sulphur hexafluoride filled microbubbles with phospholipid shells, was reconstituted in normal saline at a ratio of 1 : 5 ml, and agitated for complete dissolution. Each rabbit underwent a bolus injection of 0.3 ml of SonoVue solution followed by 5 ml flush of saline in bolus via the margin ear vein through a three-way tube. A dynamic image of CEUS was acquired until the end of the contrast agent wash-out phase. All investigations were performed in the standardized method by the same ultrasound physician [16]. CEUS of VX2 tumors was performed before MVA, and CEUS of IRB and residual tumors was performed 30 min after MVA in each rabbit under anesthesia. All dynamic images were reviewed by two ultrasound physicians to record the enhancement patterns of VX2 tumors before MVA, IRB, and residual tumors after MVA. The enhanced areas of the VX2 tumor, IRB, residual tumor, and normal liver parenchyma were segmented manually as sampling areas, and one sample from each sampling area was taken as regions of interest (ROIs) to develop time-intensity curves (TIC). Perfusion parameters were then quantitatively estimated using the software available on the GE LOGIQ E9 ultrasound machine, by which quantitative parameters were generated from curve fitting formula

(1) $$\begin{array}{c} F\left(t\right)=A\left(1-\exp\left[-kt\right]\right)+B, \end{array}$$

where *A* is the plateau value as an estimate of the regional blood volume, *k* is the replenishment rate as an estimate of blood velocity, *A*^∗^*k* is an estimate of blood flow, *B* is the baseline, and *t* is the time [9].

### 2.5. Histopathological Examination

Following the completion of the CEUS, liver biopsies of residual tumors, IRB, and junctional areas were gathered using an 18-G automatic biopsy gun (Bard Biopsy System, Tempe, AZ, USA) under anesthesia. Biopsy sample selections were guided by CEUS following a second injection of SonoVue. All biopsy specimens were removed for pathological examination using hematoxylin-eosin (HE) staining and VEGFR-2 immunohistochemical staining. Each rabbit was euthanized with an intravenous injection of sodium pentobarbital (100 mg/kg) after the biopsy.

### 2.6. Statistical Analyses

Statistical analysis was performed using SPSS. Continuous variables were presented as mean ± standard deviation (SD) and analyzed using Student's *t*-test, where a *p* value of <0.05 was considered to indicate a statistically significant difference.

## 3. Results

All rabbits successfully tolerated VX2 tumor inoculation and MVA operation. No mortality was observed during the research process.

### 3.1. Ultrasonic Findings of VX2 Tumors

**Figure 2:**
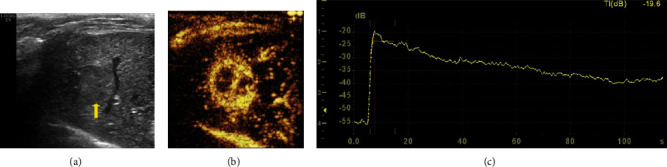
Ultrasound image of VX2 liver tumor in rabbits. A gray-scale ultrasound (a) showed a single sphere-alike homogeneous hypoechoic nodular with a clear boundary (arrow). The CEUS (b) and time-intensity curve (c) of VX2 liver tumor showed a characteristic “rapid wash-in and wash-out” vascular perfusion pattern.

At 14 days posttumor inoculation, the rabbit liver tumors showed sphere-shaped or ellipsoid homogeneous hypoechoic nodules with a clear boundary and without capsule echo. CEUS showed marked peripheral enhancement of the VX2 tumors at the early arterial phase followed by quick wash-out of contrast agents. CEUS time-intensity curves of VX2 tumors also showed a characteristic “rapid wash-in and wash-out” vascular pattern (Figure 2).

**Figure 3:**
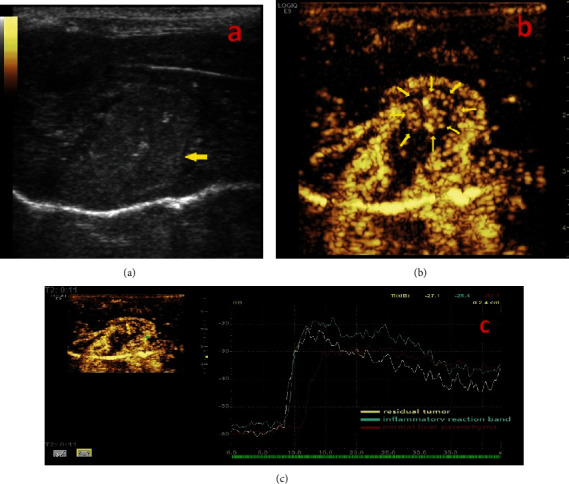
CEUS of residual tumor after MVA. Two-dimensional ultrasound (a) showed a single heterogeneous hyperechoic nodular (arrow) following MVA. The CEUS (b) and time-intensity curve (c) of the tumor area demonstrated a focal enhancement during the arterial phase indicating part of the tumor nodule remained active (arrow), which meant the animal model of inflammatory response zone coexisting with residual tumor was successfully established.

After partial ablation of tumor nodules, no residual tumors could be identified using the gray-scale ultrasound. In CEUS, the residual tumors can be detected clearly with a significant focal hyperenhancement during the arterial period, while the IRB surrounding the ablation foci simultaneously showed an annular-shaped enhancement with a consistent thickness (Figure 3).

**Figure 4:**
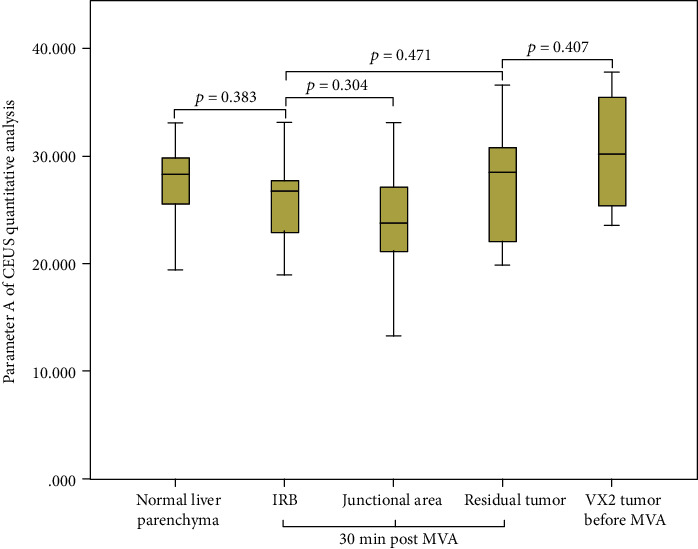
Regional blood volume estimated by parameter *A* of CEUS quantitative analysis. IRB: inflammatory reaction band; MVA: microwave ablation.

**Figure 5:**
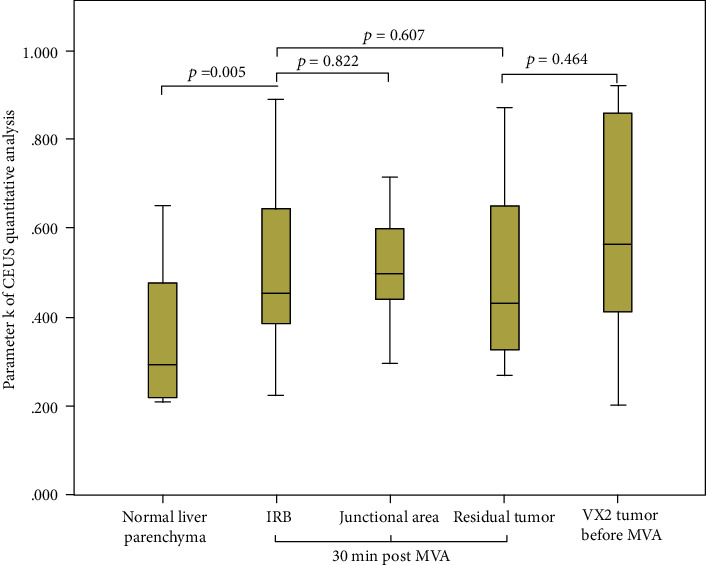
Regional blood velocity estimated by parameter *A* of CEUS quantitative analysis.

**Figure 6:**
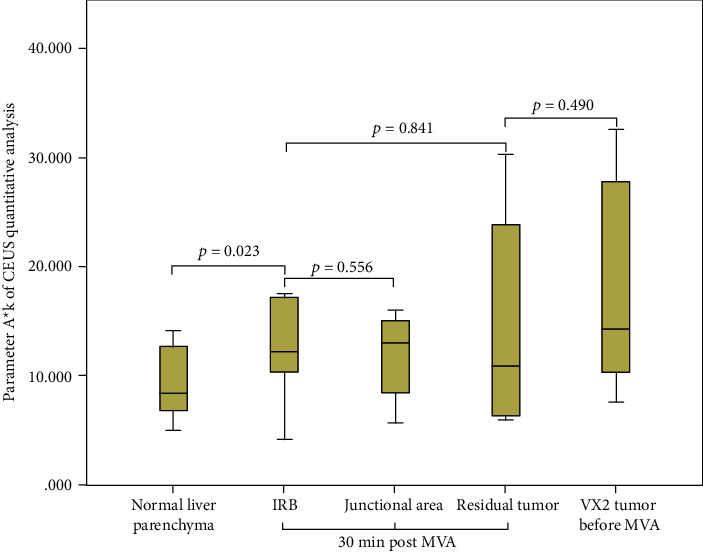
Regional blood flow estimated by parameter *A* of CEUS quantitative analysis.

The results of parameters derived from time-intensive curves of CEUS are presented in Table 1. The quantitative analysis of CEUS revealed that there was no statistically significant difference between the IRB and residual tumor in regional blood volume, velocity, and blood flow estimated by parameters *A*, *k*, and *A*^∗^*k*, respectively (*p* = 0.471, 0.670, 0.841, respectively). No statistical difference was found between the IRB and the junctional area in the regional blood volume, velocity, and blood flow (*p* = 0.304, 0.822, 0.556, respectively). No statistically significant difference was revealed between the residual tumor and VX2 tumor before the ablation in regional blood volume, velocity, and blood flow (*p* = 0.407, 0.464, 0.490, respectively). There was a statistically significant difference between the IRB and normal liver parenchyma in regional blood velocity and blood flow (*p* = 0.005, 0.023, respectively) (Figures 4, 5, and 6).

### 3.2. Pathological Findings

**Figure 7:**
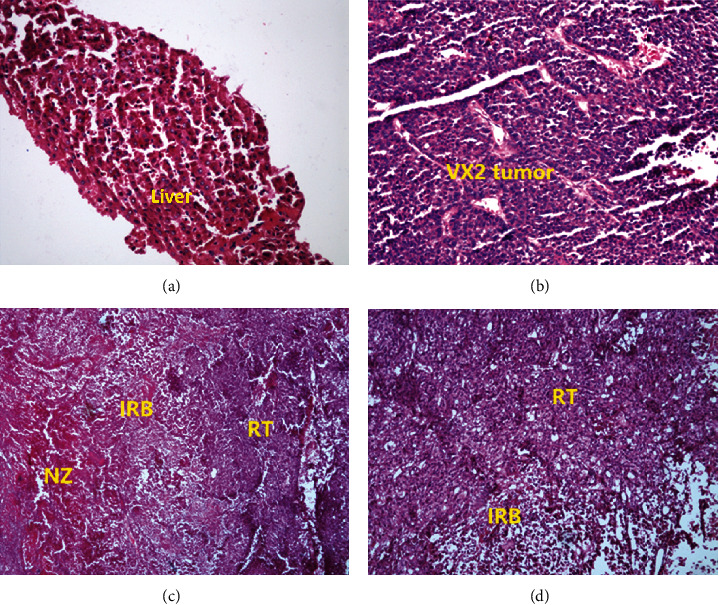
Histopathological examination image (HE staining). (a) Normal liver parenchyma. (b) VX2 tumor before microwave ablation shows the typical cancer cells with malignant morphology. (c, d) Residual tumor (RT) and inflammatory reaction band (IRB) coexist after ablation, IRB reveals blood vessel dilation, and necrosis zone (NZ) shows coagulation necrosis.

The normal liver parenchyma revealed normal tissue structure and arrangement of hepatocytes, the size and shape of hepatocytes were equal, and the nucleus was homogenous. The VX2 tumor area showed irregular cancer nests with a higher prevalence of small blood vessels and capillaries. The tumor cells demonstrated an irregular shape, disordered arrangement, rich cytoplasm, and enlarged nuclei. IRB revealed visible blood vessel dilation and inflammatory cell infiltration, such as granulocytes, plasma cells, and lymphocytes. The necrosis zone showed coagulative necrosis including nucleus contraction and dissolution (Figure 7).

### 3.3. VEGFR-2 Immunohistochemical Findings

**Figure 8:**
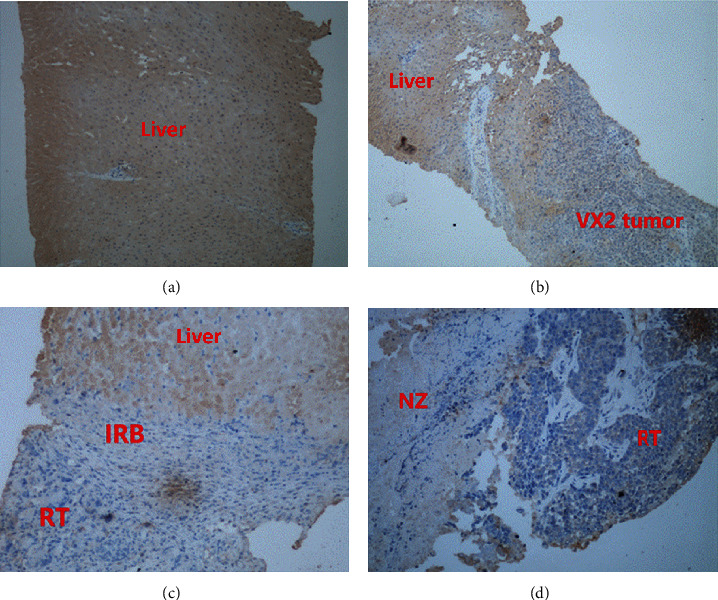
VEGFR-2 immunohistochemical examination image. (a) Normal liver parenchyma shows nonspecific staining. (b) VX2 tumor before microwave ablation shows positive staining. (c, d) Residual tumor (RT) and inflammatory reaction band (IRB) coexist after ablation, RT shows positive staining, IRB shows weak positive staining, and the necrosis zone (NZ) shows negative staining.

The normal liver parenchyma showed nonspecific VEGFR-2 staining, while the VX2 tumors before ablation and residual tumors after ablation both showed positive staining. The necrosis zone showed negative staining (Figure 8).

## 4. Discussion

CEUS can provide real-time contrast agent wash-in and wash-out kinetics, and the use of time-intensity curve (TIC) can provide a real-time quantitative analysis of local contrast enhancement [17]. Compared with CT and MRI, ultrasound has the unique advantages of nonionizing radiation and equipment portability. At present, CEUS has been widely used in the exploration and localization of liver tumors, interventional ablation guidance, and therapy evaluation [11, 18]. Although highly echoic bubbles caused by thermal ablation could interfere with the ultrasound evaluation of the ablation zone, the harmonic contrast enhancement mode used in CEUS can significantly reduce the interference caused by nonresonant gases. In our study, CEUS images were not affected after 30 minutes postablation. Using CEUS, we found that all VX2 tumors exhibited the same enhancement pattern: rapid wash-in followed by rapid wash-out including residual tumors following MVA, which is the hallmark of HCC.

In this study, no statistical differences were found by CEUS quantitative analysis between IRB vs. residual tumor, IRB vs. junctional area, and residual tumor vs. VX2 tumor before ablation. There was a statistically significant difference between the IRB and normal liver parenchyma in regional blood velocity and blood flow as estimated by parameter *k* and *A*^∗^*k* of CEUS quantitative analysis (*p* = 0.005, 0.023, respectively). These results suggest that MVA had no significant effect on the blood flow of residual tumor, but IRB was immediately formed post-MVA, and the blood flow pattern in IRB increased significantly as compared to normal liver parenchyma. The IRB blood flow pattern was also similar to tumor tissue. Additionally, this study also showed that the use of CEUS quantitative parameters could not early identify residual tumors within the IRB.

In CEUS, the IRB and residual tumors surrounding the ablation foci showed significantly higher enhancement during the arterial period. However, the IRB region showed an annular-shaped enhancement with consistent thickness, while the residual tumor showed a focal hyperenhancement. Therefore, when the residual tumor is large, the focally enhanced residual tumor and the annularly enhanced IRB can be distinguished. When the residual tumor is small, however, the residual tumor nodule and the annular IRB spatially overlap, making it impossible to determine if a residual tumor exists in the highly contrast-enhanced area until the hyperemia zone disappears or the residual tumor grows, which occurs later at a median time of 9.9 months reportedly [19].

Therefore, CEUS can detect residual tumors only when they protrude from the annular IRB region immediately post-MVA [20]. Consequently, based on this study, conventional CEUS is limited in detecting residual tumor after MVA for HCC. However, CEUS can accurately assess the extent of the necrotic region postablation, so it is necessary to confirm that the necrotic area completely covers the tumor completely.

After the thermal ablation, the annular-shaped IRB was characterized by an acute inflammatory reaction, the dilation of the marginal blood vessels in the ablation zone, the permeability of the basement membrane of the local capillary, the proliferation of erythrocyte exudation, and acute inflammatory cell aggregation. In addition, because of the tumor angiogenesis and the resulting intratumoral hypoxic microenvironment, tumor endothelial cells are stimulated to be in a continuous and highly proliferative state, resulting in a series of increase of specific molecular expression, including vascular endothelial growth factor receptor 2(VEGFR-2) [18, 21]. Our study also revealed a difference in the VEGFR-2 expression between the residual tumor and IRB postablation. Therefore VEGFR-2-targeted CEUS may have a great potential for early detection of residual tumor postthermal ablation of HCC and is worthy of further investigation in future studies.

## 5. Conclusions

MVA had no significant effect on residual tumor hemodynamics, and the blood flow in IRB increased significantly as compared to normal liver parenchyma and resembles tumor hemodynamic patterns. Conventional CEUS is limited in identifying residual tumor post-MVA for HCC. CEUS quantitative parameters could not early identify the possible residual tumor within the IRB immediately postablation. CEUS can detect residual tumors immediately postablation only when they protrude from the annular-shaped IRB. In addition, VEGFR-2-targeted CEUS may have a great potential for detecting residual tumor after thermal ablation of HCC.

## Figures and Tables

**Table 1:** Results of parameters of time-intensive curves of CEUS.

Parameters	Normal liver parenchyma	30 min post-MVA			VX2 tumor before MVA
		IRB	Junctional area	Residual tumor	
*A*	27.623 ± 3.916	26.012 ± 4.056	24.058 ± 5.332	27.538 ± 5.521	30.048 ± 4.840
*k*	0.355 ± 0.152	0.522 ± 0.195	0.533 ± 0.164	0.496 ± 0.210	0.589 ± 0.227
*A*∗*k*	9.402 ± 3.113	13.869 ± 6.413	13.123 ± 5.946	14.403 ± 8.264	17.968 ± 8.729

IRB: inflammatory reaction band; MVA: microwave ablation.

## Data Availability

All relevant data are within this manuscript, and all data are fully available without restriction.
